# Dual RNA-Seq Unveils the Role of the *Pseudomonas plecoglossicida fliA* Gene in Pathogen-Host Interaction with *Larimichthys crocea*

**DOI:** 10.3390/microorganisms7100443

**Published:** 2019-10-11

**Authors:** Yujia Sun, Pin Nie, Lingmin Zhao, Lixing Huang, Yingxue Qin, Xiaojin Xu, Jiaonan Zhang, Qingpi Yan

**Affiliations:** 1 Fisheries College, Key Laboratory of Healthy Mariculture for the East China Sea, Ministry of Agriculture, Jimei University, Xiamen 361021, China; sunyj37@mail2.sysu.edu.cn (Y.S.); 200561000132@jmu.edu.cn (L.Z.); lixinghuang@jmu.edu.cn (L.H.); yxqin@jmu.edu.cn (Y.Q.); xiaojinxu@jmu.edu.cn (X.X.); 2 School of Marine Science and Engineering, Qingdao Agricultural University, Qingdao 266109, China; pinnie@qau.edu.cn; 3 Key Laboratory of Special Aquatic Feed for Fujian, Fujian Tianma Technology Company Limited, Fuzhou 350308, China; jolma66@163.com

**Keywords:** pathogen-host interaction, *Larimichthys crocea*, *Pseudomonas plecoglossicida*, *fliA*, dual RNA-seq

## Abstract

In the present study, *Larimichthys crocea* and *Pseudomonas plecoglossicida* were selected as a host-pathogen interaction model for teleosts and prokaryotic pathogens. Five shRNAs were designed and synthesized to silence the *fliA* gene, all of which resulted in pronounced reductions in *fliA* mRNA; the mutant strain with the best silencing efficiency of 92.16% was chosen for subsequent analysis. A significant decrease in motility, intracellular survival and escape was observed for the *fliA*-RNAi strain of *P. plecoglossicida*, whereby silencing of the *fliA* gene led to a 30% decrease in mortality and a four-day delay in the onset of infection in *L. crocea*. Moreover, silencing of *P. plecoglossicida fliA* resulted in a significant change in both the pathogen and host transcriptome in the spleens of infected *L. crocea*. Kyoto Encyclopedia of Genes and Genomes (KEGG) analysis of pathogen transcriptome data showed that silencing *fliA* resulted in downregulation of 18 flagellum-related genes; KEGG analysis of host transcriptome data revealed that infection with the *fliA*-RNAi strain caused upregulation of 47 and downregulation of 106 immune-related genes. These pathogen-host interactions might facilitate clearance of *P. plecoglossicida* by *L. crocea*, with a significant decrease in *fliA*-RNAi *P. plecoglossicida* strain virulence in *L. crocea*.

## 1. Introduction

Flagella are an important structure in bacteria and are widely recognized to be involved in their pathogenesis [1,2]. For example, flagella contribute to the virulence of prokaryotic pathogens by promoting adherence [3,4] and the formation of bacterial biofilms [5,6], exporting virulent factors via the type III secretion system [7,8], and activating inflammation via the recognition of Toll-like receptor 5 [9,10]. More than 50 genes are involved in the synthesis of flagella, and they are expressed in a strictly regulated and hierarchical manner [11].

σ^28^, which is encoded by the *fliA* gene, plays a positive regulatory role in flagellar assembly [12,13]. σ^28^ has been documented to regulate flagellar synthesis in *Escherichia coli* [14], *Campylobacter jejuni* [15], *Pseudomonas aeruginosa* [16], *Salmonella typhimurium* [17] and *Vibrio cholera* [18]. Furthermore, knocking out *fliA* in *P. aeruginosa* resulted in reduced motility, with decreased colonization in the intestines of mice because flagella were not synthesized [19]. In addition, the *fliA* mutant strain of *Legionella pneumophila* exhibits reduced motility, weakened biofilm, reduced macrophage infectivity and decreased colonization potential in host cells [20]. Although *fliA* is well known for its important multiple functions, the role of *fliA* in host-pathogen interactions remains unknown, partly due to the limitations of research technology.

The infection process is a fierce battle between the host and pathogen, in which both must strive for success [21]. To win this life-and-death struggle, both pathogen and host must mobilize all available resources, and all changes will be reflected in their respective transcriptome profiles [22]. Dual RNA-seq offers the ability to monitor host-pathogen RNA expression profiles simultaneously [23,24,25,26,27].

*Pseudomonas plecoglossicida* is known to cause epidemics in cultured fish, such as ayu (*Plecoglossus altivelis*) [28], large yellow croaker (*Larimichthys crocea*) [29,30] and rainbow trout (*Oncorhynchus mykiss*) [31]. The pathogenic mechanism of *P. plecoglossicida* has attracted considerable attention, and several virulence genes have been recognized and explored [32,33]. In our previous research, the transcriptomes of *P. plecoglossicida* in the spleens of infected *L. crocea* were sequenced at 1, 2, 3 and 4 days post-infection (dpi); these data have been deposited in the NCBI database (SRP176599). *fliA* was noted for its significant upregulation during the entire infection process, and it was hypothesized to play an important role in the host-pathogen interaction.

To explore the role of *fliA* of *P. plecoglossicida* in the host-pathogen interaction, the gene was knocked down by RNAi, and phenotypic differences between *fliA*-RNAi and wild type strains of *P. plecoglossicida* were analyzed. In addition, dual RNA-seq was performed using the spleens of *L. crocea* infected by the *fliA*-RNAi strain or wild type strain of *P. plecoglossicida* as material, and the data were subjected to a series of bioinformatics analyses. The present study constitutes a new attempt to explore the role of a single gene in the host-pathogen interaction by integrating dual RNA-seq and RNAi.

## 2. Results

### 2.1. Construction of the fliA-RNAi Strain of P. plecoglossicida

**Figure 1:**
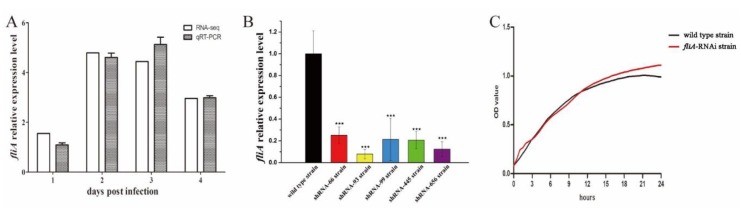
Construction of the *fliA*-RNAi strain of *P. plecoglossicida*. (**A**) Relative expression of *fliA* at 1, 2, 3 and 4 days post-infection. Each data comes from three samples. (**B**) Relative expression of *fliA* in five mutant strains of *P. plecoglossicida*. Each data comes from three independent experiments. (**C**) growth curve of *P. plecoglossicida*. Each data point comes from 12 technical repeats. ★★★ *p* ≤ 0.001.

Figure 1A shows the RNA-seq and qRT-PCR results of *P. plecoglossicida fliA* gene expression in the spleen of *L. crocea* during the infection process. Compared with the in vitro culture counterpart, the *fliA* gene of was upregulated in the infected spleen at all four sampling times, with the highest expression recorded at 2 dpi. The results of qRT-PCR were consistent with those of RNA-seq.

According to qRT-PCR results, all five shRNAs designed in this study had a silencing effect on *fliA* expression in *P. plecoglossicida* (Figure 1B). The strain containing pCM130/tac-*fliA*-shRNA-93 exhibited the best *fliA*-silencing efficiency (92.16%) and was chosen as the *fliA*-RNAi strain for further analysis.

A growth curve was generated to determine whether *fliA* affects growth. Although *fliA* was silenced, no significant difference in growth rate between the *fliA*-RNAi strain and wild type strain of *P. plecoglossicida* was found when cultured in vitro at 28 °C (Figure 1C).

### 2.2. The Effect of fliA on Motility and Intracellular Survival of P. plecoglossicida

**Figure 2:**
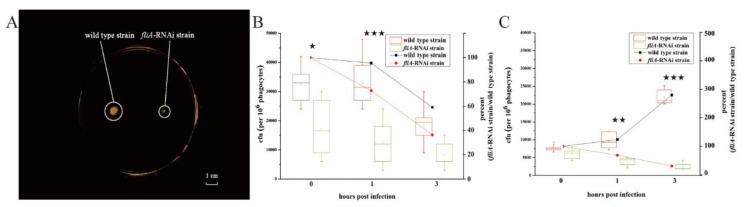
Characterization of the *fliA*-RNAi strain and wild type strain of *P. plecoglossicida*. (**A**) Motility. (**B**) Survival rate in phagocytes. (**C**) Escape rate from phagocytes. The right y-axis in Figure 2B,C represent the percent of pathogen cfu number of fliA-RNAi strain group to wild type strain group. Each data point comes from three samples. ★ *p*≤ 0.05; ★★ *p* ≤ 0.01; ★★★ *p* ≤ 0.001.

The soft agar plate test showed that the colony diameters of the wild type strain were considerably larger than those of the *fliA*-RNAi strain of *P. plecoglossicida*; in contrast, the colony diameters of the *fliA*-RNAi strain were smaller than those of the wild type strain (Figure 2A).

After a 3-h intracellular survival assay, the number of cells of both strains of *P. plecoglossicida* was significantly reduced. The wild type strain was reduced by 40.91% and the *fliA*-RNAi strain by 63.64%, the number of *fliA*-RNAi cells was significantly reduced compared with wild type (Figure 2B). Conversely, after a 3-h intracellular escape assay, the number of wild type cells increased to 280%, though the number of *fliA*-RNAi cells was reduced to 30.95%. The escape ability of the *fliA*-RNAi strain was significantly lower than that of the wild type strain (Figure 2C).

### 2.3. The Effect of fliA on P. plecoglossicida Virulence

**Figure 3:**
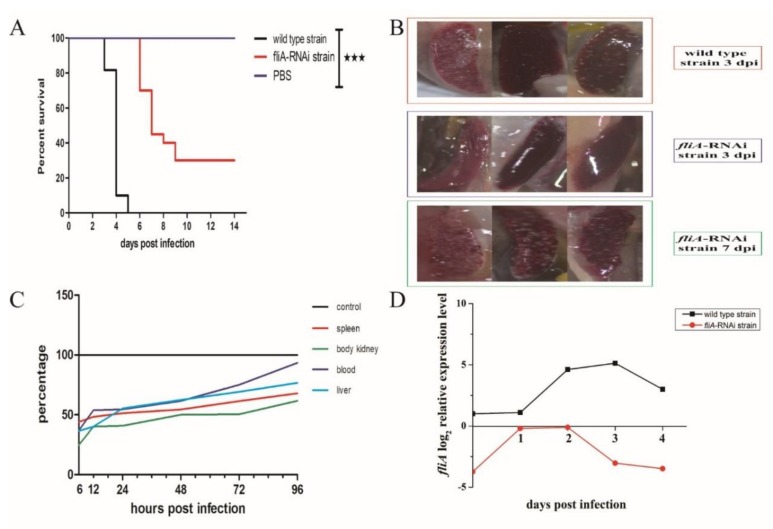
Virulence of the wild type strain and *fliA*-RNAi strain of *P. plecoglossicida*. (**A**) Survival curve of *L. crocea* infected with the wild type strain and *fliA*-RNAi strain of *P. plecoglossicida*. (**B**) Appearance of spleens of infected *L. crocea.* The upper pictures are spleens infected with the wild type strain of *P. plecoglossicida*, the middle and lower pictures are spleens infected with the *fliA*-RNAi strain of *P. plecoglossicida*. (**C**) Spatial and temporal distribution of the *fliA*-RNAi strain of *P. plecoglossicida* compared to the wild type strain. Each data point comes from three samples. (**D**) Relative expression of *fliA* of *P. plecoglossicida* in *L. crocea* spleens. Each data point comes from three samples. ★★★ *p* ≤ 0.001.

Compared with counterparts infected with the wild type strain of *P. plecoglossicida*, infection of *L. crocea* with the *fliA*-RNAi strain resulted in a delay in the time of death by 3 days, and the survival rate increased by 30% (Figure 3A).

As piscine spleens infected with *P. plecoglossicida* are usually covered with abundant white spots [21], dead fish were examined for appearance of spleens. The spleens of *L. crocea* exhibited the typical appearance, with numerous white spots, at 3 days after infection by wild type *P. plecoglossicida*, though the same appearance did not appear until 7 days after infection by the *fliA*-RNAi strain (Figure 3B).

We also assessed whether the bearing capacity of the two strains in fish organs was altered, and the difference in distribution of *P. plecoglossicida* between *L. crocea* infected with the *fliA*-RNAi and wild type strains was determined by qRT-PCR. At 6 h post-infection, the relative quantity of the *fliA*-RNAi strain in each tissue was lowest, that is, less than half of the quantity of the wild type strain, though the relative quantity of the *fliA*-RNAi strain increased gradually with the extension of infection time. Moreover, the relative quantity of the *fliA*-RNAi strain was higher in the blood than in other tissues (Figure 3C).

qRT-PCR was also employed to probe the dynamic expression of *fliA* in the two strains of *P. plecoglossicida* during infection. Expression of *fliA* was upregulated during infection with the wild type strain, increased to a peak at 2 dpi, and then decreased gradually. Similarly, the expression of *fliA* in the *fliA*-RNAi strain increased at 1 and 2 dpi and then decreased gradually. Overall, expression of *fliA* in both strains was higher in vivo than in vitro, and expression of *fliA* in the *fliA*-RNAi strain was lower than that in the wild type strain throughout the study (Figure 3D).

Briefly, silencing *fliA* in *P. plecoglossicida* resulted in increased survival, delayed time of death and appearance occurrence, reduced quantity distribution in *L. crocea* and decreased expression of *fliA* throughout the infection process.

### 2.4. The Effects of fliA on the Transcriptome of Host and Pathogen

The base distributions of spleen and in vitro-cultured *P. plecoglossicida* were balanced, and the unknown base N distribution was lower than that of any other base and in a reasonable range (Figures S1 and S2). The average distribution of base error rates was less than 0.1%, a reasonable range (Figures S3 and S4). To reduce the complexity of data and illustrate the relationship between samples, principal component analysis was carried out, and the result showed that the repeatability of samples was satisfactory (Figures S5 and S6). Twenty-three differentially expressed genes (DEGs) of the host and pathogen transcriptomes were randomly selected and verified by qRT-PCR, and the results were consistent with the transcriptome results (Figure S7).

**Figure 4:**
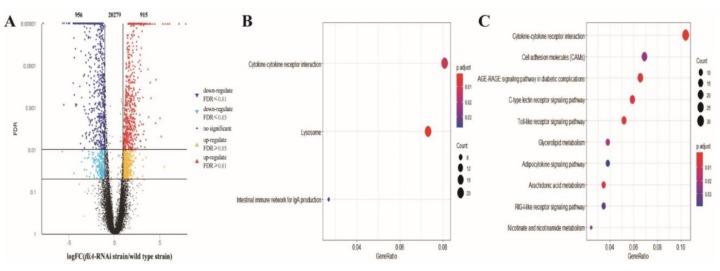
Global characterization of the host transcriptome in the spleen of *L. crocea* infected by *P. plecoglossicida*. (**A**) Volcano plot of all genes of the spleen. X-axis: the fold change values of genes of the *fliA*-RNAi strain infection group/wild type strain infection group. Y-axis: statistical test value (false discovery rate (FDR)), higher values represent more significant differences. Each dot represents a particular gene: red dots denote significantly upregulated genes, blue dots significantly downregulated genes and black dots genes with non-significant differences. (**B**) KEGG enrichment of upregulated genes. (**C**) KEGG enrichment of downregulated genes. In B and C, the X-axis shows the GeneRatio, whereby a higher value indicates more genes enriched in the pathway; the Y-axis shows the enriched pathways, and the more red the dot is, the more significant is the pathway.

A total of 22,150 genes were mapped for the host transcriptome. Compared to infection by the wild type strain of *P. plecoglossicida*, infection by the *fliA*-RNAi strain resulted in 956 host genes being downregulated and 915 being upregulated in the spleen; the other 20279 genes showed no significant difference (Figure 4A). According to the KEGG database, 47 upregulated DEGs were enriched in three immune-related KEGG pathways, and adjusted *p*-values from high to low indicated involvement of the cytokine-cytokine receptor interaction pathway, lysosome pathway and intestinal immune network and the IgA production pathway (Figure 4B). The lysosome pathway was enriched 19 upregulated genes, encoding glycosidase, protease, phosphatase, sulfatase and lysosomal membrane proteins. Additionally, 106 downregulated DEGs were enriched in 10 KEGG pathways, with 51 DEGs enriched in six immune-related KEGG pathways. Adjusted *p*-values from high to low showed involvement of the cytokine-cytokine receptor interaction pathway, cell adhesion molecule pathway, AGE-RAGE signaling pathway in the diabetic complications pathway, C-type lectin receptor signaling pathway, Toll-like receptor signaling pathway, glycerolipid metabolism pathway, adipocytokine signaling pathway, arachidonic acid metabolism pathway, RIG-I-like receptor signaling pathway and nicotinate and nicotinamide metabolism pathway (Figure 4C).

**Figure 5:**
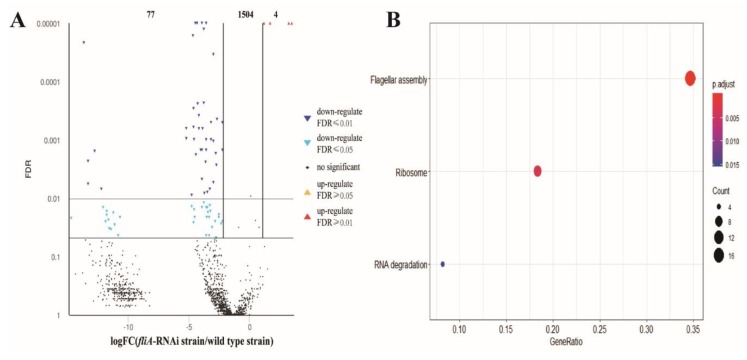
Global characterization of the pathogen transcriptome in the spleen of *L. crocea* infected by *P. plecoglossicida*. (**A**) Volcano plot of all genes of *P. plecoglossicida*. X-axis: the fold change values of genes of the *fliA*-RNAi strain infection group/wild type strain infection group. Y-axis: the statistical test value (false discovery rate (FDR)), whereby a higher value represents a more significant difference. Each dot represents a particular gene: red dots denote significantly upregulated genes, blue dots significantly downregulated genes and black dots genes with non-significant differences. (**B**) The X-axis shows the GeneRatio, whereby a higher value indicates more genes enriched in the pathway; the Y-axis shows the enriched pathways, and the more red the dot is, the more significant is the pathway.

For the pathogen transcriptome, a total of 1585 genes were mapped. At 2 dpi, 1504 genes were not significantly different, 77 genes were downregulated, and four genes were upregulated in fish infected by the *fliA*-RNAi strain of *P. plecoglossicida* compared to the pathogen transcriptome in the spleens of fish infected by the wild type strain (Figure 5A). According to the KEGG database, 30 downregulated DEGs were enriched in three KEGG pathways, including the flagellar assembly pathway, ribosome pathway and RNA degradation pathway, as presented by *p-values* from high to low (Figure 5B).

### 2.5. The Relationship between DEGs of P. plecoglossicida

**Figure 6:**
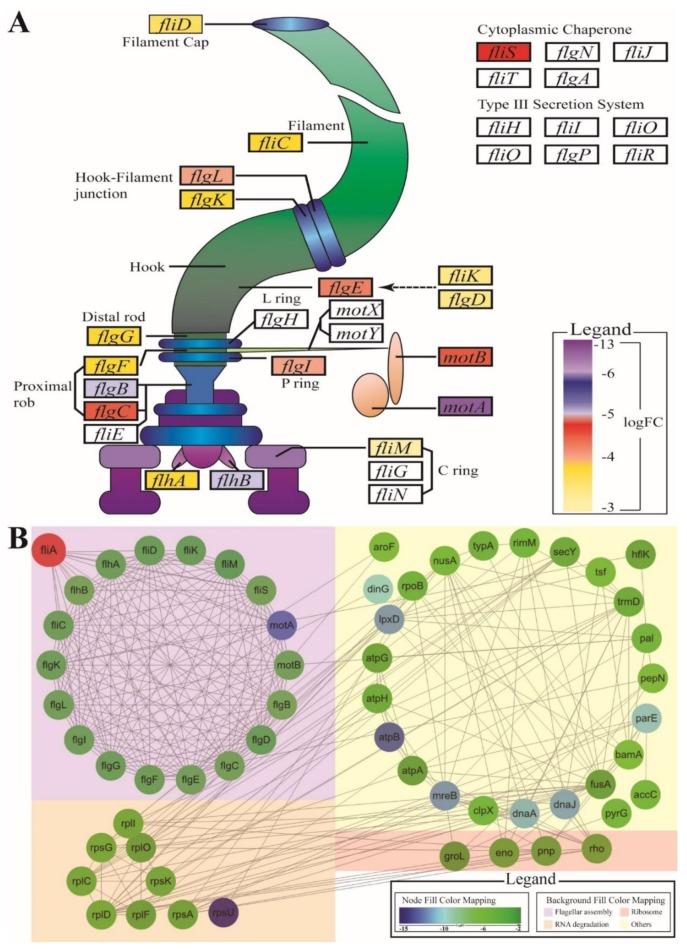
Changes in gene expression in the flagellar assembly pathway and related pathway of *P. plecoglossicida*. (**A**) Visualization of the flagellar assembly pathway. Genes with different colors indicate different logFC values. (**B**) The relationship between flagellar assembly genes and other genes. Genes range in color from green to blue, whereby a bluer color indicates a greater downregulation of expression. The *fliA* gene is red. The purple background indicates the flagellar assembly pathway; the orange background indicates the RNA degradation pathway; the red background indicates the ribosome pathway; the yellow background indicates other differentially expressed genes.

RNAi of *fliA* had a great effect on the expression of genes in the flagellar assembly pathway at 2 dpi. Compared with the group infected by the wild type strain, expression of 18 genes in the flagellar assembly pathway of *P. plecoglossicida* was downregulated, which included 52.94% of genes in the flagellar assembly pathway and 73.91% of flagellar structure genes (Figure 6A). Conversely, no flagellar assembly pathway genes were found to be upregulated.

In addition to genes in the flagellar assembly pathway, some genes in other pathways were downregulated by *fliA* RNA. According to the network constructed by GENEMANIA, *fliA* controls the flagellar assembly pathway by regulating expression of 13 genes, and *fliA* regulates non-flagellar genes through genes of the flagellar assembly pathway; flagellar genes indirectly regulate genes of the ribosome pathway, the RNA degradation pathway and other pathways (Figure 6B).

## 3. Discussion

Several genes have been identified to be involved in the pathogenesis of *P. plecoglossicida* [29,32,33,34] and other aquatic pathogens [35,36]. Accumulating evidence indicates that flagella are an important structure for pathogenic bacteria [37,38,39]. Prior to this work, *fliA* had been described as an essential gene for flagellar synthesis and pathogenesis [19,20]; therefore, it is necessary to explore the role of *fliA* in the host-pathogen interaction of *P. plecoglossicida*.

In the present study, all five shRNAs caused a significant silencing effect on *fliA*, though with different efficiencies. The shRNA targeting 93–113 bp downstream of the translation initiation site of the *fliA* gene had the best silencing efficiency, of 92.16%, and the effect persisted at all sampling times. The result of this silencing established the foundation for further research examining the role of *fliA* post-infection. Compared to infection of *L. crocea* by the wild type strain, *fliA*-RNAi strain infection resulted in a 3-day delay in the time of death, a 30% reduction in mortality and a 4-day delay of appearances in the spleen. The spleen is a major immune organ that is both specific and non-specific in fish [40,41]. In addition, the spleen is the organ displaying typical appearances [42] and maximum amounts of bacteria [34]. Thus, it may be inferred that the spleen is the ideal tissue for researching the interaction of *L. crocea* and *P. plecoglossicida*.

Overall, the transcriptome reflects the complete physiological condition of an organism [33]. In a strain of *P. aeruginosa* lacking *fliA*, eight genes were downregulated and four were upregulated in the flagellar assembly pathway after culture in LB medium [19]. In the present study, silencing *fliA* in *P. plecoglossicida* resulted in the identification of 18 genes downregulated in the flagellar assembly pathway (52.94%), but no gene was observed to be upregulated. Although many studies have investigated the role of *fliA* in flagellar synthesis, this report is the first to describe that *fliA* affects so many flagellar genes during infection. The results indicate that *fliA* plays a critical role in flagellar assembly, especially in the host. Flagellar and ribosome assembly involve the two largest biomacromolecular complexes in the cell, and they coordinate via the energy cycle [43]. In the present study, the ribosome and RNA degradation pathways of *P. plecoglossicida* were also downregulated as a result of *fliA* RNAi, suggesting that *fliA* plays a more complex role in the regulation of pathogen transcription in the host than previously known.

Dual RNA-seq technology is beginning to be employed to simultaneously monitor global changes between host and pathogen [23,26,27]. In the present study, during infection, the deficient strain of *fliA* showed blocked flagellar synthesis, and expression of Toll-like receptor 5 (*tlr5*) was decreased, as expected. *tlr5* specifically recognizes flagellin [10,44] and can induce expression of several proinflammatory cytokine genes, such as *il-1β* and *tnfα* [45,46], the latter of which is activated by arachidonic acid in Kupper cells [47]. In the present study, *fliA* RNAi in *P. plecoglossicida* resulted in downregulation of *il-1β* and *tnfα* expression in the host, as well as *il-6*, *il-8* and *il-12*, which are known to be regulated by Toll-like receptor 4 (*tlr4*) [48,49,50,51]. In addition, the arachidonic acid metabolism pathway was downregulated, indicating a negative regulatory mechanism between *tnfα* and arachidonic acid. Moreover, a coexpression relationship has been documented for arachidonic acid metabolism and nicotinamide metabolism, which may explain the downregulation of nicotinate and nicotinamide metabolism pathways [52]. The nicotinamide and glycerolipid metabolism pathways are both damaged in chronic progressive heart failure, though the mechanism has not been full elucidated [53]. Flagella facilitate the survival of bacteria within macrophages or their escape from macrophages [2,54]. In the present study, RNAi of *fliA* resulted in the reduced ability of *P. plecoglossicida* to survive and escape from phagocytes as well as a decrease in motility. Moreover, *fliA* RNAi led to upregulation of some host genes related to lysosomes, including glycosidase, protease, phosphatase and sulfatase, which facilitate host clearance of pathogens.

## 4. Materials and Methods

### 4.1. Bacterial Strains and Culture Conditions

*P. plecoglossicida* NZBD9 was isolated from the spleen of a *L. crocea* individual with “Visceral White Spot Disease” [42]. The *E. coli* DH5α strain was purchased from TransGen Biotech (Beijing, China). The bacteria were routinely cultured in Luria Bertani (LB) medium at 18 °C (*P. plecoglossicida*) and 37 °C (*E. coli*). LB medium containing 1 μg/mL tetracycline was used to select the strain containing the plasmid pCM130/tac.

### 4.2. Construction of the RNAi Strain

pCM130/tac [34] was used to construct the *P. plecoglossicida* RNAi strain according to a previously described method [55,56]. Five siRNA sequences targeting the *fliA* gene were designed by BLOCK-iTTM RNAi Designer (http://rnaidesigner.thermofisher.com/rnaiexpress/setOption.do?designOption=shrna&pid=708587103220684543) and synthetized by Shanghai Generay Biotech Co., Ltd. (Shanghai, China; Table S1). First, the pCM130/tac vector was linearized using the restriction enzymes *NsiI-HF* and *BsrGI-HF* (New England Biolabs, Ipswich, MA, USA) and ligated to annealed shRNA with T4 DNA ligase. Second, the recombinant plasmid was transformed into *E. coli* DH5a by heat shock, extracted using EasyPure Plasmid MiniPrep Kit (TransGen Biotech, Beijing, China) and electroporated into *P. plecoglossicida*. Each strain was cultured to an OD_600_ 0.5 ± 0.1, and the level of *fliA* mRNA was detected by quantitative real-time (qRT)-PCR for three independent experiments.

### 4.3. Quantitative Real-Time PCR (qRT-PCR)

qRT-PCR was performed using QuantStudio 6 Flex (Life Technologies) as previously described [57]. All primer sequences used in the present study are provided in Table S2. Each qRT-PCR experiment was repeated five times.

### 4.4. Growth Curve Determination

The wild type strain or *fliA*-RNAi strain of *P. plecoglossicida* was cultured in LB medium at 28 °C until the OD reached 0.4~0.6 and then diluted to 0.2 ± 0.01. Next, 50 μL aliquots of diluted bacterial suspension was added to twelve wells of microtiter plate which preload 150 μL sterile LB medium and incubated at 28 °C. The values of OD_600_ of each well were read using a SYNERGY H1 microplate reader (BioTec, Dorset, UK).

### 4.5. Swimming Motility Assay

The swimming motility assay was performed using plates half-filled LB medium with 0.3% agar. The wild type and *fliA*-RNAi strains of *P. plecoglossicida* were incubated until the value of OD_600_ reached 0.4~0.6; the culture medium was then diluted to an OD of 0.3 ± 0.01, and 1 μL was inoculated onto the centre of each plate [37,58]. The assay was technical repeated three times.

### 4.6. Intracellular Survival and Escape Assay

Healthy *L. crocea* individuals were purchased from Ningde (Fujian, China) and anaesthetized with 4-ethyl-amino-benzocaine before head kidney sampling. The tissues were pushed through a 100-mesh nylon screen and suspended in L-15 medium (Biological Industries, Kibbutz Beit- Haemek, Israel) containing 100 IU streptomycin/penicillin (S/P)/mL and 2% fetal calf serum (FCS). The tissues were transferred to a 200-mesh nylon screen and centrifuged at 400× *g* for 20 min, and 2 mL L-15 medium was added. The cell suspension was layered onto a 34%/51% discontinuous Percoll (Amersham Pharmacia Biotech, New York, NY, USA) density gradient with a syringe and centrifuged at 400× *g* for 20 min at 4 °C. Next, the cells in the layer above the 34%/51% interface were collected, washed twice with phosphate-buffered saline (PBS) and resuspended in L-15 medium with 10% FCS, 100 IU S/P/mL. The cells were then incubated at 28 °C for 3 h, after which non-adherent cells were removed by washing with L-15, and the monolayers were collected. The cell suspension was added to 2 × 10^6^ cells/mL in L-15 medium with 10% FCS and 100 IU S/P/mL and transferred to six-well plates at 1.5 mL/well. The pathogen was added in a volume of 1.5 mL to each plate in a ratio of 1 cell to 100 pathogen cells, which were washed twice with PBS. The cells were infected with the wild type or *fliA*-RNAi strain incubated with 250 μg/mL tetracycline or ofloxacin at 28 °C for 20 min, and infected cells were washed with PBS, centrifuged at 400× *g* and 20 min, and resuspended in 3 mL L-15 medium containing 10% FCS and 100 IU S/P/mL. The 3-mL cell solution was divided into three parts on average for three checkpoints. Each part was centrifuged at 400× *g* for 20 min, and the supernatant was diluted 10 times and inoculated onto plates. The infected cells at the bottom of the tube were lysed with ddH_2_O, diluted 100 times and inoculated onto plates to determine the survival rate [59]. The assay was repeated three times.

### 4.7. L. crocea Infection Experiments

All fish experiments were executed strictly following the recommendations of ‘National Institutes of Health Guide for the Care and Use of Laboratory Animals’. The animal protocols were conducted strictly according to the Animal Ethics Committee of Jimei University (Acceptance NO JMULAC201159, date of approval: 20 December 2011).

Weight-matched healthy *L. crocea* were maintained at 18 °C to adapt to the experimental temperature for one week. The wild type and *fliA*-RNAi strains of *P. plecoglossicida* were incubated at 18 °C until reaching an OD_600_ value of 0.4~0.6 and were then centrifuged and diluted with PBS.

One infection experiment was carried out for survival rate assay. 120 *L. crocea* were randomly divided into six groups, three groups for wild type strain of *P. plecoglossicida* challenge; the other three groups for RNAi strain challenge. Each fish was jnjected intrapleurally with 10^4^ colony-forming units of the wild type or *fliA*-RNAi strain of *P. plecoglossicida* per gram fish (cfu/g); 60 fish injected with PBS were used as a negative control. *L. crocea* mortality was recorded every day.

Another infection experiment was carried out for sampling. 60 fish were challenged with wild type or *fliA*-RNAi strain of *P. plecoglossicida* as described above. For spatial and temporal distribution assays, three random fish from both wild type and *fliA*-RNAi strain infection groups were used to sample the spleen, liver, trunk kidney and blood at 6, 12, 24, 48, 72 and 96 hpi. For the dual RNA-seq assay, nine fish from both wild type and *fliA*-RNAi strain infection groups were used to sample the spleen at 2 dpi. Three spleens were pooled as one independent sample.

### 4.8. DNA and RNA Extraction

Genomic DNA was extracted from the spleens, livers and trunk kidneys of infected *L. crocea* using EasyPure Marine Animal Genomic DNA Kit (TransGen Biotech, Beijing, China). Genomic DNA was extracted from the blood using EasyPure Blood Genomic DNA Kit (TransGen Biotech, Beijing, China).

Total RNA was isolated from the spleens, livers, blood and trunk kidneys using the TRIzol reagent (Invitrogen, Carlsbad, CA, USA), and the coexisting DNA was digested by Turbo DNA-free DNase (Ambion, Austin, TX, USA). The quality of the RNA obtained was assessed using an Agilent 2100 Bioanalyzer (Agilent Technologies, Santa Clara, CA, USA), and rRNA was removed by Ribo-Zero rRNA Removal Kit (Epicentre, Madison, WI, USA). cDNA synthesis was conducted with TransScript All-in-One First-Strand cDNA Synthesis SuperMix for PCR (TransGen Biotech, Beijing, China).

### 4.9. Illumina Sequencing

The cDNA libraries of spleens were amplified by Phusion DNA polymerase (New England Biolabs, Ipswich, MA, USA), and an Agilent 2100 Bioanalyzer (Agilent Technologies, City of Santa Clara, CA, USA) was employed to validate the library quality. Finally, sequencing was performed by Majorbio Biotech Co., Ltd. (Shanghai, China) using the Illumina HiSeq 4000 sequencing platform.

### 4.10. Processing and Mapping of Reads

Filtering and quality control of the raw Illumina reads were performed by SeqPrep (https://github.com/jstjohn/SeqPrep), Sickle (https://github.com/najoshi/sickle) and Fastx-Toolkit (http://hannonlab.cshl.edu/fastx_toolkit/) with default settings. The clean data were mapped to the NB2011 strain genome of *P. plecoglossicida* (NCBI RefSeq accession numbers: NZ_ASJX00000000.1) by Bowtie2 [60]. Mapped reads were mapped to the *P. plecoglossicida* genome and the *L. crocea* genome.

### 4.11. Analysis of Differential Gene Expression

Identification of differentially expressed genes was performed using the R package edgeR (version 3.10.2) [61]. The condition met the thresholds |log2^fold change^| ≥ 1 and false discovery rate (FDR) < 0.05.

### 4.12. KEGG Enrichment Analysis

KEGG enrichment analysis was performed using the R package ‘clusterProfiler’ [62]. The thresholds pvalueCutoff ≤ 0.05 and qvalueCutoff ≤ 0.05 were applied to identify differentially expressed KEGG pathways.

### 4.13. Statistical Analyses

All data are presented as the mean ± standard deviation (SD) based on at least three independent experiments. Dunnett’s test was used to calculate different experimental data using SPSS 17.0 software (Chicago, IL, USA). GraphPad Prism software was employed for survival rate analysis.

### 4.14. Data Access

The RNA sequencing reads data were deposited in the GenBank SRA database under the accession number SRP183207.

## 5. Conclusions

**Figure 7:**
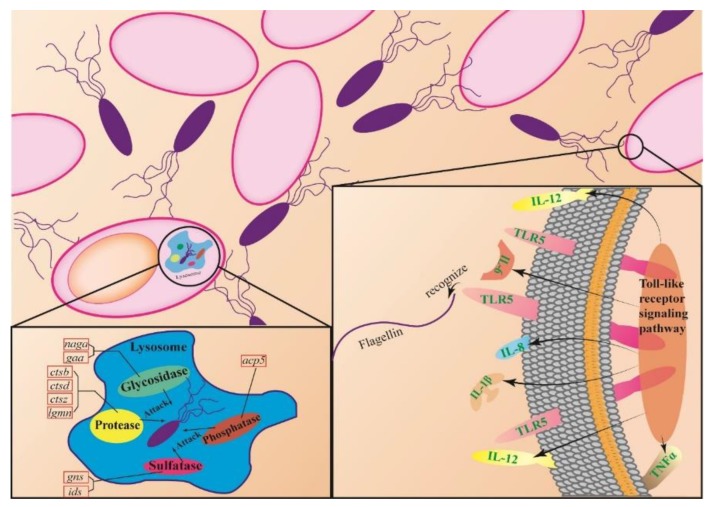
Role of *P. plecoglossicida fliA* during interaction with *L. crocea*. The schematic diagram of host pathogen interaction was drawn based on the experimental results of this paper and the literatures.

In summary, silencing of the *fliA* gene inhibited the synthesis and assembly of *P. plecoglossicida*’s flagella, enabling *P. plecoglossicida* to be engulfed by *L. crocea* phagocytes and leading to upregulation of phagocytosis-related genes; however, the relationship between the decrease in the number of extracellular pathogens and the downregulation of several immune pathway genes needs to be explained by further experiment (Figure 7). These host-pathogen interactions facilitate removal of *P. plecoglossicida* by *L. crocea*, and a significant decrease in the virulence to *L. crocea* of the *fliA*-RNAi strain of *P. plecoglossicida* was observed.

## Supplementary Materials

Supplementary materials can be found at https://www.mdpi.com/2076-2607/7/10/443/s1. Figure S1. Eukaryotic base percentage composition reads. Figure S2. Prokaryotic base percentage composition reads. Figure S3. Eukaryotic distribution of mean error. Figure S4. Prokaryotic distribution of mean error. Figure S5. Eukaryotic PCA analysis. Figure S6. Prokaryotic PCA analysis. Figure S7. Verification dual transcriptome data by qRT-PCR. Table S1. shRNA sequences used in this study. Table S2. Primer sequences used in this study.
