# Supplementary material for: Dual RNA-Seq Unveils the Role of the Pseudomonas plecoglossicida fliA Gene in Pathogen-Host Interaction with Larimichthys crocea
Source: Microorganisms. 2019 Oct 11;7(10):443. doi: 10.3390/microorganisms7100443 (PMC6843279; doi:10.3390/microorganisms7100443)
Supplement: Supplementary file 1 [file microorganisms-07-00443-s001.pdf]

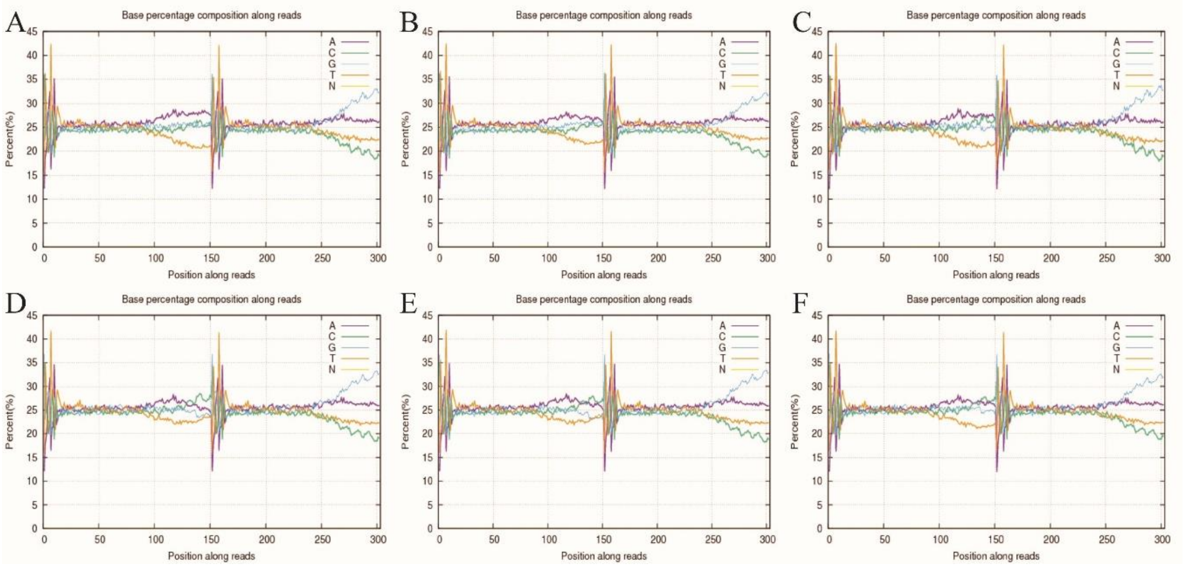

Figure S1. Eukaryotic base percentage composition along reads. Horizontal axis is base coordinates of reads, which represent the base from the 5' to the 3' end sequentially. Vertical axis is corresponding percentage, each base with different color. A is purple; C is green; G is blue; T is orange; N is yellow. But in existing high-throughput sequencing technologies, reverse transcribed into cDNA was used in 6 bp random primers can cause a few position of nucleotide composition exist certain preferences, which belongs to the normal situation. (**A**), (**B**), (**C**) are wild type strain; (**D**), (**E**), (**F**) are *fliA*-RNAi strain.

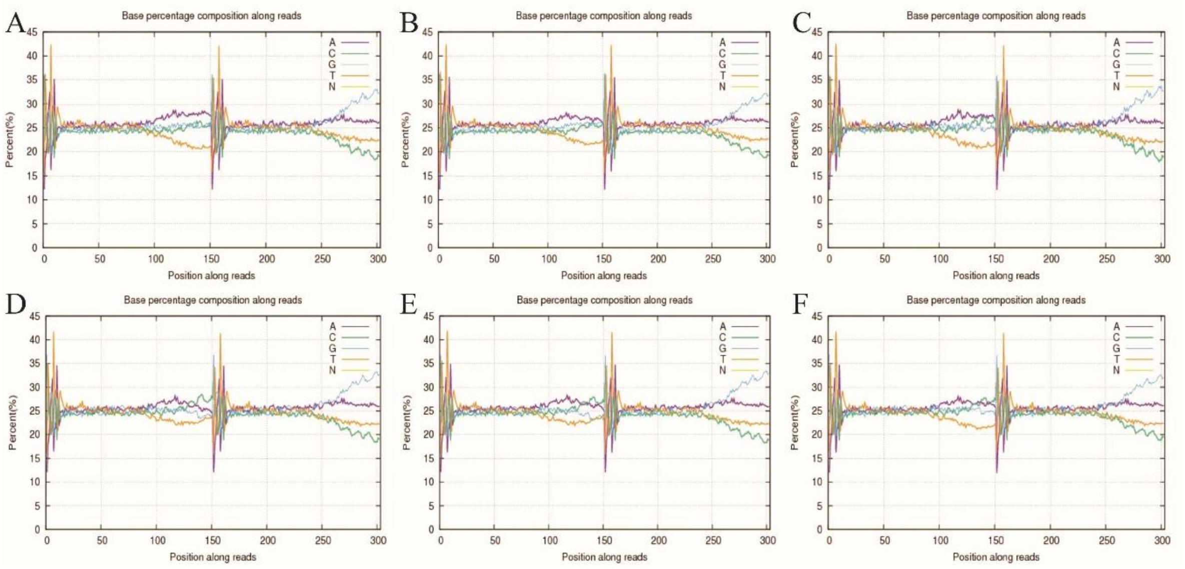

Figure S2. Prokaryotic base percentage composition along reads. Horizontal axis is base coordinates of reads, which represent the base from the 5' to the 3' end sequentially. Vertical axis is corresponding percentage, each base with different color. A is purple; C is green; G is blue; T is orange; N is yellow. But in existing high-throughput sequencing technologies, reverse transcribed into cDNA was used in 6 bp random primers can cause a few position of nucleotide composition exist certain preferences, which belongs to the normal situation. (**A**), (**B**), (**C**) are wild type strain; (**D**), (**E**), (**F**) are *fliA*-RNAi strain.

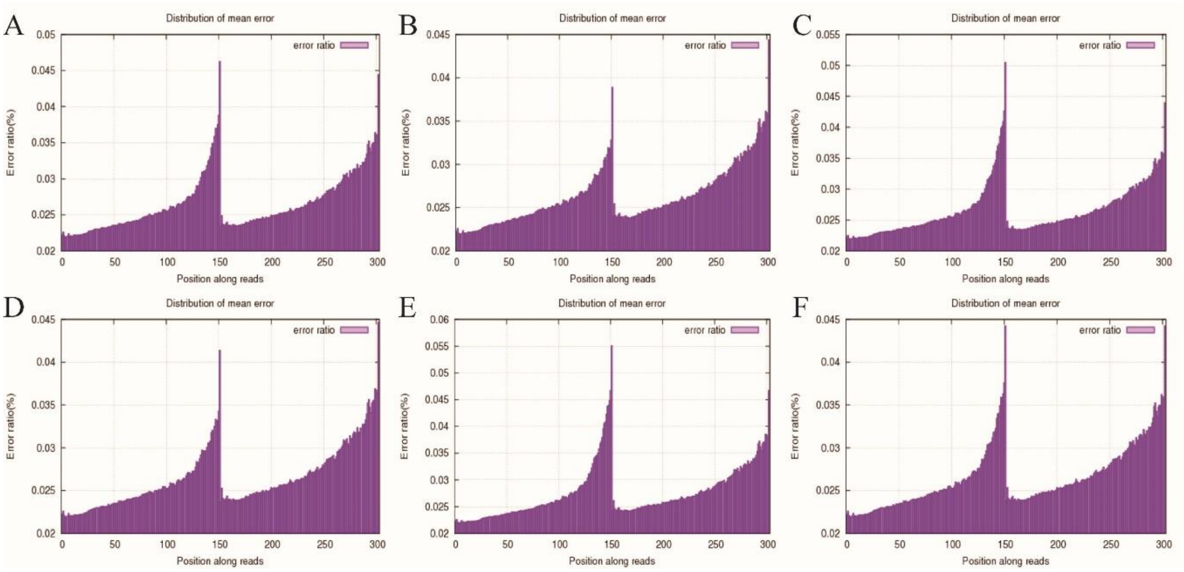

Figure S3. Eukaryotic distribution of mean error. Distribution of mean error. Horizontal axis is base coordinates of reads, which represent the base from the 5' to the 3' endsequentially. Vertical axis is the average base error rate of all reads at the site (%). The purple shadow in the graph corresponds to theaverage value of the base error rate, which reflects the distribution of base error rate in the sequencing reads, and the acceptable rangeof the average base error rate is less than 0.1%. (**A**), (**B**), (**C**) are wild type strain; (**D**), (**E**), (**F**) are *fliA*-RNAi strain.

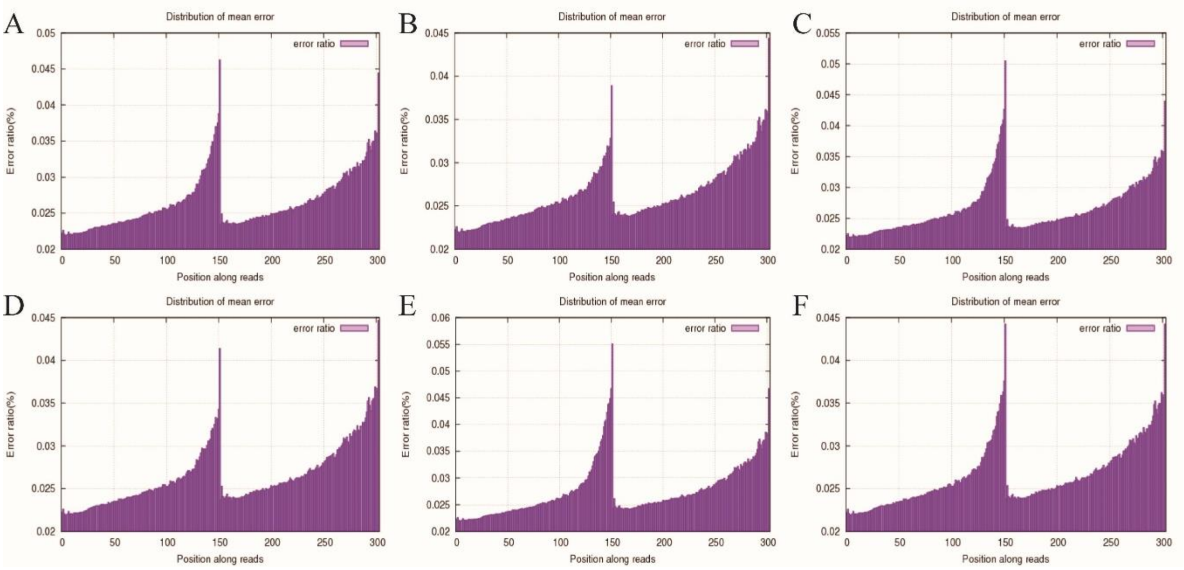

Figure S4. Prokaryotic distribution of mean error. Distribution of mean error. Horizontal axis is base coordinates of reads, which represent the base from the 5' to the 3' endsequentially. Vertical axis is the average base error rate of all reads at the site (%). The purple shadow in the graph corresponds to theaverage value of the base error rate, which reflects the distribution of base error rate in the sequencing reads, and the acceptable rangeof the average base error rate is less than 0.1%. (**A**), (**B**), (**C**) are wild type strain; (**D**), (**E**), (**F**) are *fliA*-RNAi strain.

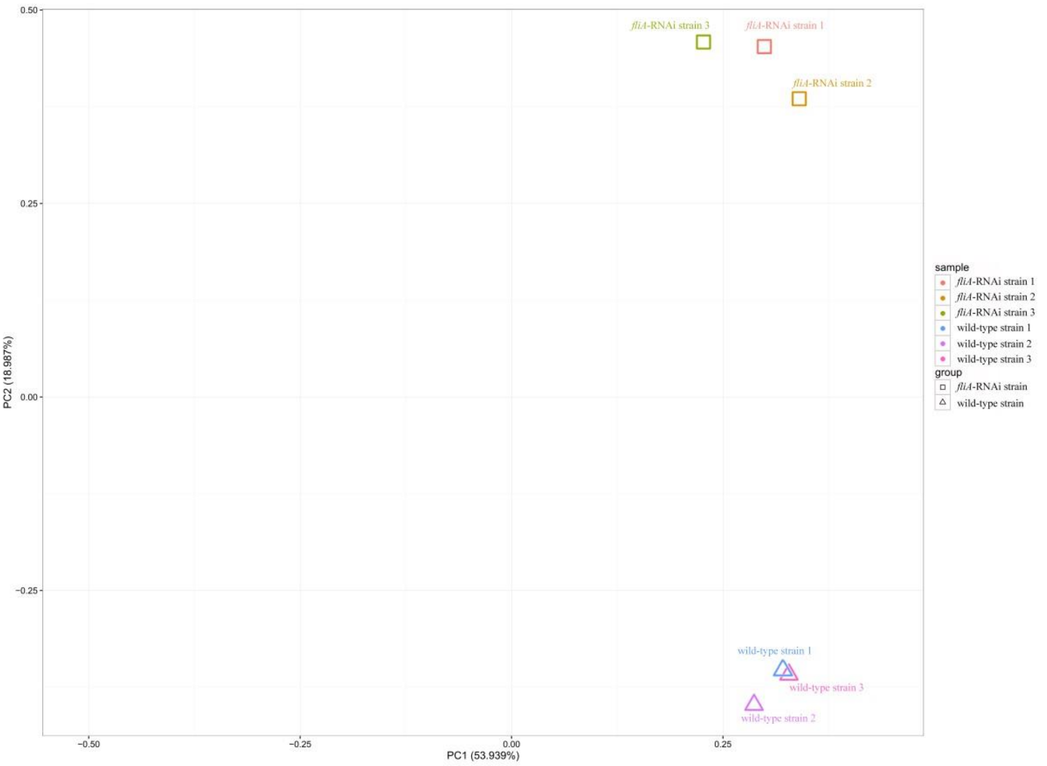

Figure S5. Eukaryotic PCA analysis. Principal component analysis(PCA) can reduce the complexity of data, and dig deep the relation between sample size and variation. The basic principle is that diverse samples have different measurement, PCA is to find out the main factors of observed value differences, considering all the factors are combined and sort according to importance. Usually the tiny factors are ignored, which play a role of simplify the data. For two or three principal components axis graphed, which can see the distance of the relationship between each sample, including visual effect of clusters groups.

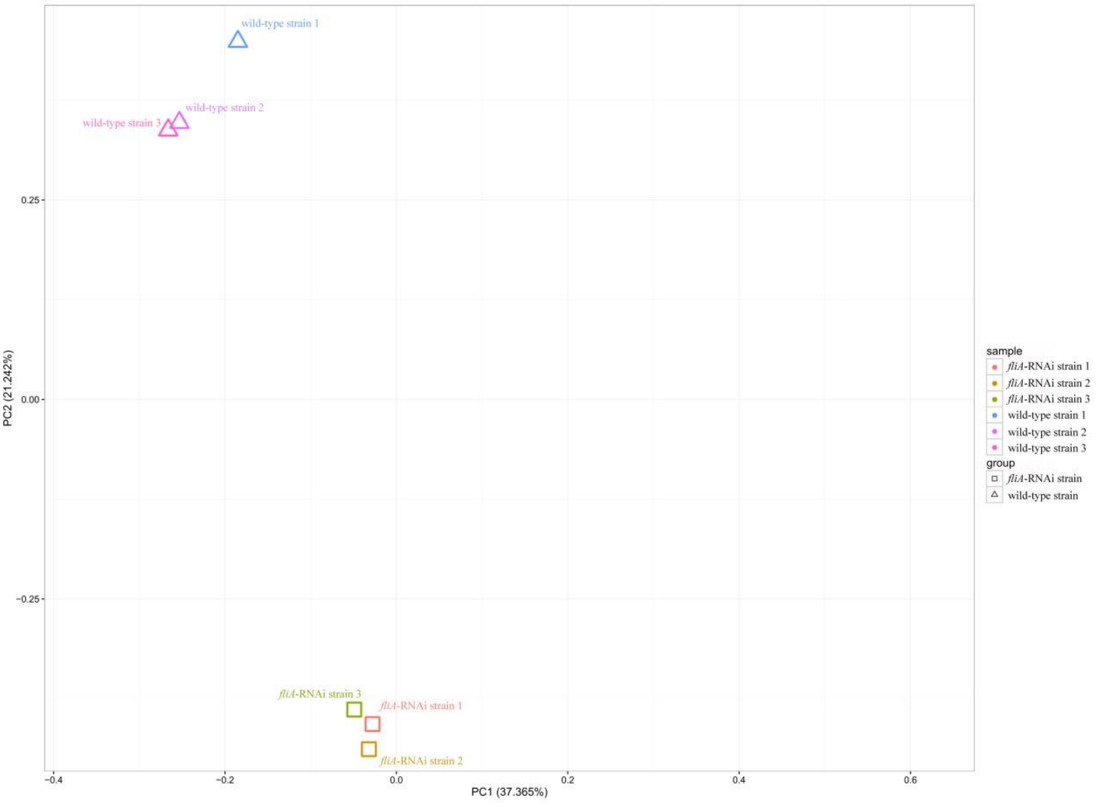

Figure S6. Prokaryotic PCA analysis. Principal component analysis(PCA) can reduce the complexity of data, and dig deep the relation between sample size and variation. The basic principle is that diverse samples have different measurement, PCA is to find out the main factors of observed value differences, considering all the factors are combined and sort according to importance. Usually the tiny factors are ignored, which play a role of simplify the data. For two or three principal components axis graphed, which can see the distance of the relationship between each sample, including visual effect of clusters groups.

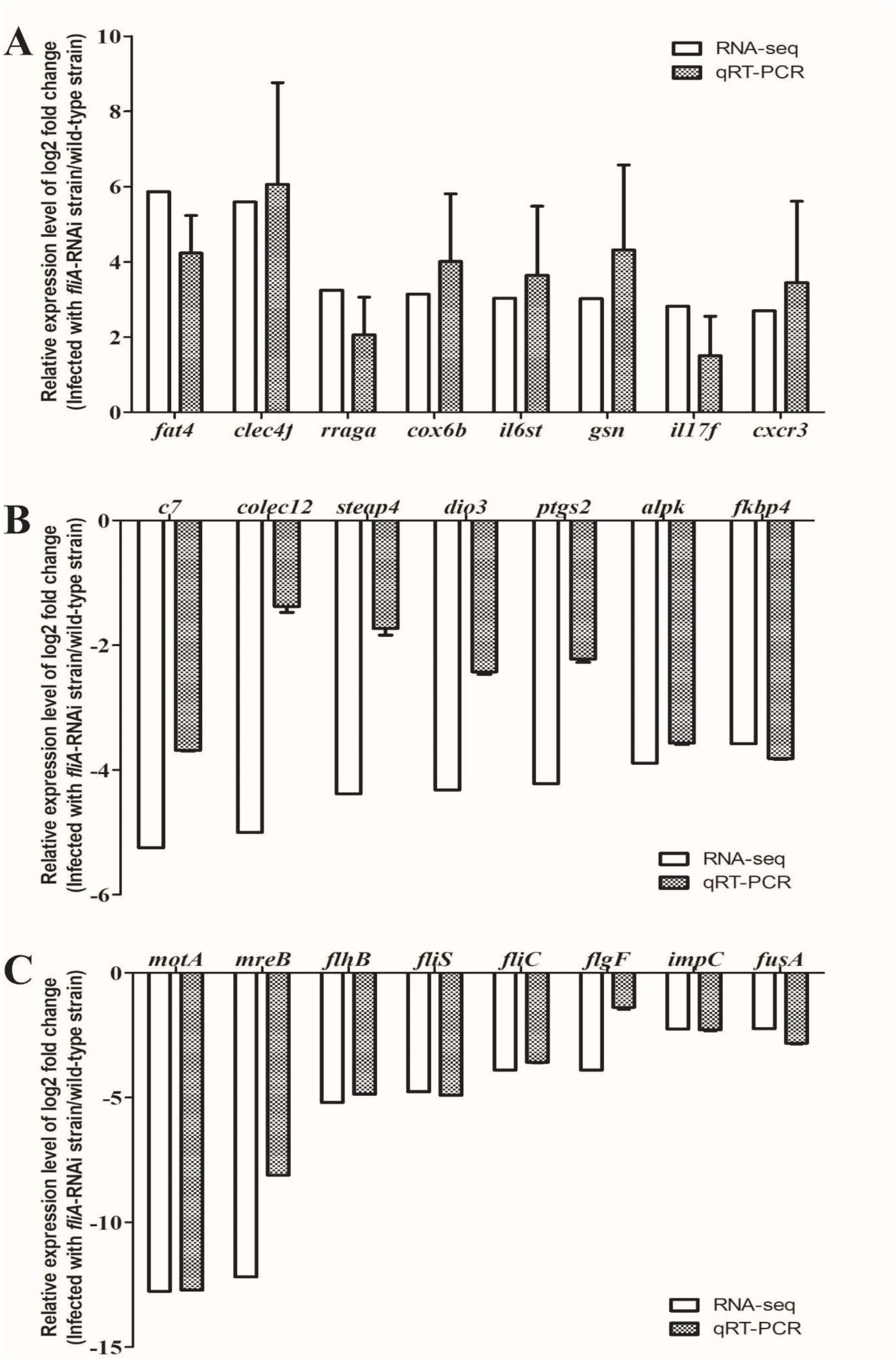

Figure S7. Verification transcriptome data by qRT-PCR. Relative changes in abundance of selected genes. qRT-PCR was performed by triple technical repetions. White bars: RNA-seq; Grid bars: qRT-

PCR. A: up-regulated genes in eukaryotic transcriptome; B: down-regulated genes in eukaryotic transcriptome; C: down-regulated genes in prokaryotic transcriptome.

Table S1. List of shRNA sequence used to silence *fliA* expression in present study.

Name	Sequence(5′-3′ orientation)
shRNA-66	TGCGTTGGTTGATGGACTACCTTTCAAGAGAAGGTAGTCCATCAACCAACGCTTTTTT<br>GTACAAAAAAAGCGTTGGTTGATGGACTACCTTCTCTTGAAAGGTAGTCCATCAACCAACGCATGCA
shRNA-93	TGGTCAAGCGCATTGTCAATCATTCAAGAGATGATTGACAATGCGCTTGACCTTTTTT<br>GTACAAAAAAAGGTCAAGCGCATTGTCAATCATCTCTTGAATGATTGACAATGCGCTTGACCATGCA
shRNA-99	TGCGCATTGTCAATCAGCTATCTTCAAGAGAGATAGCTGATTGACAATGCGCTTTTTT<br>GTACAAAAAAAGCGCATTGTCAATCAGCTATCTCTCTTGAAGATAGCTGATTGACAATGCGCATGCA
shRNA-445	TGCCATCGAAAGCCTCGATACATTCAAGAGATGTATCGAGGCTTTCGATGGCTTTTTT<br>GTACAAAAAAAGCCATCGAAAGCCTCGATACATCTCTTGAATGTATCGAGGCTTTCGATGGCATGCA
shRNA-656	TGGGTCTGTCAGTTGAGTAAGCTTCAAGAGAGCTTACTCAACTGACAGACCCTTTTTT<br>GTACAAAAAAAGGGTCTGTCAGTTGAGTAAGCTCTCTTGAAGCTTACTCAACTGACAGACCCATGCA

Table S2. List of qRT-PCR primers used to determine gene expression changes in present study.

Gene name	Forward primer sequence(5′-3′ orientation)	Reverse primer sequence sequence(5′-3′ orientation)	Source
16S	TCAGTATCAGTCCAGGTGGTCG C	CGTTACCGACAGAATAAGCACC G	(1)
β-actin	GACCTGACAGACTACCTCATG	AGTTGAAGGTGGTCTCGTGGA	(2)
gyrB	TGCTGAAGGACGAGCGTTCG	ATCATCTTGCCGACAACAGC	(3)
fliA	CGAGGGTTTCTTCAGAGCGGTA C	GCACCAGGCCAATTTCTTTAAG AT	This study
C7	GCCGAACCCGTTTACTTG	TCATCGCAGGATATGGAC	This study
il1b	TAAGACGGAAGTTTGGAT	AAGCGTTTCACTTTCTGTAC	This study
colec12	GGAGAATGGAAATGGGTG	GGAGCAGGGAACATCGTA	This study
steap4	GAAGGTGCTGGTGGATGT	TCTACTGGCGTCTGAGGG	This study
dio3	GCACCGCTGTTTGGAGGA	CGGCGTTGGATGAGTTGTC	This study
ptgs2	TGATGTTCGCATTCTTCG	TTGCCTTTCCAGGTTGTC	This study
alpk	GGAATGGGTAACTGATGT	GAATGGGAACTGAAACTG	This study
fkbp4	CAAGAAGACCTACGCCAATA	CTCCCTTTCAAATGTCACCA	This study
fat4	GGAATGGGAGAATGGAAC	ACAAGACAGAACAGGAGC	This study
clec4f	TCTGAGTGACAGCCTTGA	CCTTCCTTCTGCACGATT	This study
rraga	GCGATACGCTCTGTTGAA	ACAGCAAAGACAGCCCAC	This study
cox6b	AAAGGGTCTACAAATCAATC	TCTCCCACCACTTCAACA	This study
il16st	GGCCAAGACGACCTCTAACG	GTGCTGTGCTGCCAGTGATT	This study
gsn	GATGATGACAAGGCGGACAC	GTAGCACTCTTCGGGAGACA	This study
il17f	TAGAATCCCGTTTACCTCAG	CTTCCTCCCTCCTTTGTTAT	This study
cxcr3	ACGGACACCTTCCTCCTTCA	AACAGCCAATCCAAACACCC	This study
motA	TCTTCCTTTGCTATTGCCTGATG	TGTTTGAACGCTGGCTTGAC	This study
mreB	TCTCGCTGAACGGCGTGGTC	GATGCGCTCGGCGGTGGATT	This study
flhB	TGGTGGTCAATGTGATGGTAGT	GGTGATCGGGTTTAGCTTTT	This study
fliS	AAAGGGAAATCGCTGGAGAA	AACGGTGCAAACAGTAATCGTA T	This study
fliC	GCCTCGTTCGGTGCCAATCT	CGCTTTCCAGGGCGTAGTCG	This study
flgF	CAAGGGCGGCGAAGCCTATA	CCCGTGGCAACACCGAGACA	This study
impC	CGAACTCGTGCCAGAAACCA	CCAGGCGGCTGACCACAAAA	This study
fusA	CCGTCATCAACTCGGTCAAG	CAGGGAAGTCCATACGCTCA	This study

Supplementary references

1. Sun, Y.; Luo, G.; Zhao, L.; Huang, L.; Qin, Y.; Su, Y.; Yan, Q. Integration of RNAi and RNA-seqReveals the Immune Responses of *Epinepheluscoioides* to *sigX* Gene of *Pseudomonas plecoglossicida*. *Front. Immunol.* **2018**, *9*, 1624.
2. Zheng, W.; Liu, G.; Ao, J.; Chen, X. (2006). Expression analysis of immune-relevant genes in the spleen of large yellow croaker (Pseudosciaena crocea) stimulated with poly I: C. *Fish Shellfish Immunol*. **2006**, *21*, 414-430.
3. Izumi, S.; Yamamoto, M.; Suzuki, K.; Shimizu, A.; Aranishi, F. Identification and detection of *Pseudomonas plecoglossicida* isolates with PCR primers targeting the *gyrB* region. *J. Fish Dis.* **2007**, *30*, 391–397.
